# Mind Kills Body

**Published:** 1871-06

**Authors:** 


### MIND KILLS BODY.

For twenty years' faithful service a lady left in her will, made at Norwich, Connecticut, twenty thousand dollars to Abby Nilkey, who in less than two weeks died of excess of joy.

Within a week an estimable young lady of St. Johnsbury, Vermont, was driven to suicide by the slanderous utterance of a liar.

A man was so outraged in De Kalb, Illinois, a week ago, by a slander on his mother, that he shot the liar, who died on the spot.

Scarce a day passes in which some one does not commit suicide under depressing mental influences, the result of bodily ailments, which send bad blood to the brain; hence bodily health should be the aim and study of all.

